# Unusual presentations of thoracic disc herniation treated by thoracic epidural block: Case reports

**DOI:** 10.1097/MD.0000000000029618

**Published:** 2022-07-29

**Authors:** Min Jong Ki, Cheol Jong Woo, Yu Jin Oh, Seon Hwa Nam, A Ram Doo

**Affiliations:** a Department of Anesthesiology and Pain Medicine, Jeonbuk National University Medical School, Jeonju, South Korea; b Research Institute of Clinical Medicine of Jeonbuk National University, Biomedical Research Institute of Jeonbuk National University Hospital, Jeonju, South Korea.

**Keywords:** abdominal pain, myelopathy, radiculopathy, thoracic disc herniation

## Abstract

**Rationale:**

Herniation of the thoracic intervertebral disc (HTD) is a rare disease that accounts for <1% of all disc herniations. Physicians may make diagnostic errors due to the variable clinical features and limited experience of HTD. In this report, we present 2 unusual cases of HTD.

**Patient concerns:**

A 72-year-old woman (case 1) visited our pain clinic because of chronic abdominal discomfort with visible bulging on the left side. Atrophy of the abdominal wall muscle and quadratus lumborum was observed. The therapeutic effect of interfascial plane block to exclude the possibility of truncal neuropathy following muscular atrophy was temporary. The other patient, a 75-year-old man (case 2) complained of aggravation of previously diagnosed postherpetic neuralgia. An extension of the previously symptomatic area of the forward upper dermatome was observed. Radiofrequency treatment on the symptomatic dorsal root ganglion failed to relieve symptoms.

**Diagnoses:**

Two patients underwent magnetic resonance imaging of the spine for further evaluation. The patients were diagnosed with multilevel HTD and foraminal herniated disc, compatible with their symptoms and without myelopathy.

**Interventions:**

Two patients were conservatively treated with a fluoroscopy-guided transforaminal epidural block.

**Outcomes:**

The 2 patients experienced significant pain reduction up to 50% on a numeric rating scale after repeated treatment.

**Lessons:**

Multilevel HTD of the mid- to lower-thoracic spine may present as abdominal bulging with atrophy of the abdominal wall muscles. We also report another case of concomitant symptomatic thoracic radiculopathy from HTD and postherpetic neuralgia at the adjacent level. Thoracic transforaminal epidural block may be considered a conservative therapeutic approach for HTD.

## 1. Introduction

Herniation of the thoracic intervertebral disc (HTD) is a rare disease that accounts for < 1% of all disc herniations,^[1]^ and it occurs most frequently at the T7–T12 level.^[2–4]^ It is usually asymptomatic but may present with upper back pain or pain radiating in a dermatomal distribution, referred to as thoracic radiculopathy.^[5]^ The sensory and motor symptoms may be equally common in cases of myelopathy accompanied by spinal cord compression.^[6]^ In a recent study, Ahi and Goktan^[7]^ suggested that the neuropathic nature of chronic back pain radiating to the chest and abdomen may be highly predictive of HTD.

Radiating truncal pain and/or concomitant back pain are somewhat nonspecific presentations in several neuropathic or musculoskeletal diseases at the thoracic level, such as intercostal neuralgia, herpes zoster, diabetic truncal neuropathy, compression fractures of the thoracic spine, and entrapment syndrome of the abdominal cutaneous nerve.^[8]^ Therefore, the differential diagnosis of thoracic radiculopathy from these more prevalent diseases is complicated for physicians in clinical practice. Moreover, the exact diagnosis can often be delayed because HTD often manifests with unexpected presentations, such as abdominal or pelvic pain, which may be misdiagnosed as a gastrointestinal or even gynecological condition.^[4]^

In this report, we present 2 unusual cases of HTD. The first patient was diagnosed with multiple thoracic disc herniations at the mid- to lower-thoracic level, which manifested as abdominal discomfort and anterolateral bulging. The second patient, who had been treated for postherpetic neuralgia at the T12 level for several months, presented with aggravation and extension of a previously symptomatic area. The patient was diagnosed with HTD at the upper adjacent level. Both patients were successfully treated with transforaminal epidural block.

## 2. Case report

Written informed consent was obtained from the patients for publication of this case report.

### 2.1. Case 1

**Figure 1.**
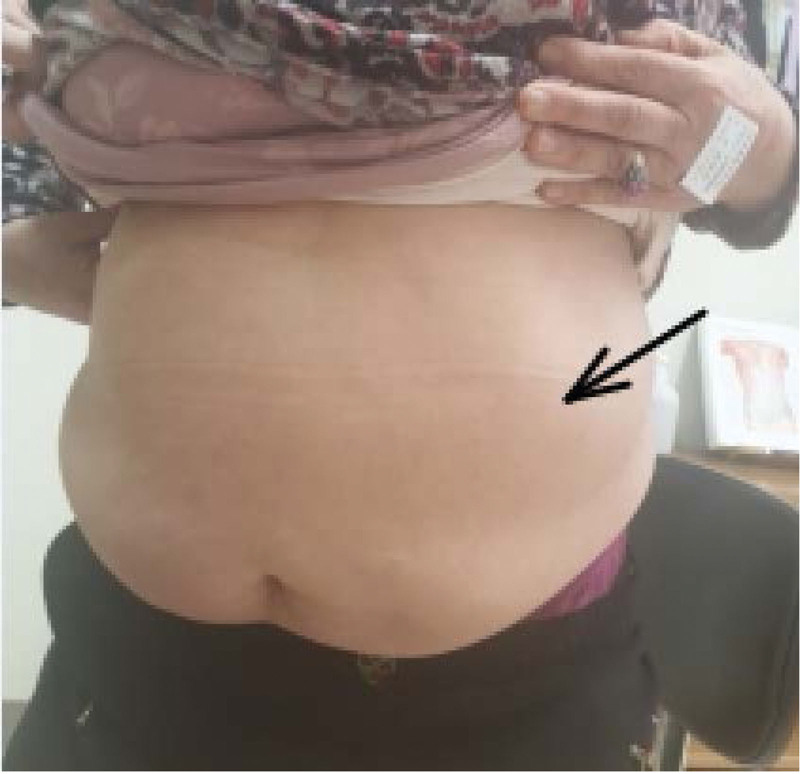
Physical examination revealing left-sided abdominal bulging in case 1.

A 72-year-old woman visited our pain clinic because of abdominal discomfort for approximately 1 year. The patient complained of aching and sore anterolateral abdominal pain with a moderate intensity of 6 on the 11-point numeric rating scale. She described her pain as unpleasant abdominal discomfort with visible bulging aggravated by meals. She also complained of shooting flank pain during positional changes or coughing. Several months before presenting at our tertiary care hospital, the patient had been examined for the possibility of various pathologies, such as gastrointestinal and genitourinary organ problems, but the laboratory and radiologic findings were nondiagnostic. The patient reported that analgesics, including a nonsteroidal anti-inflammatory drug, tramadol/acetaminophen combination, and pregabalin, did not relieve her symptoms. On physical examination, visible abdominal bulging, most prominent on the left side, was identified (Fig. 1). The patient had no remarkable sensory, neurological deficits, except for direct tenderness on the lateral abdominal wall and adjacent paraspinal muscle area.

**Figure 2.**
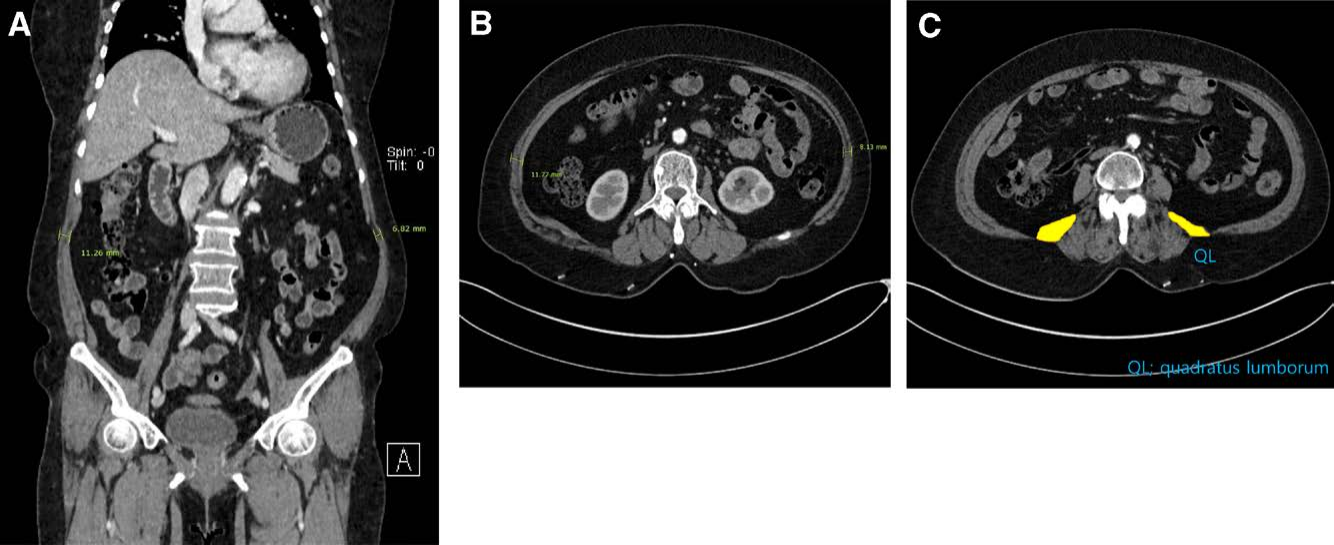
Computed tomography findings of the abdomen and pelvic cavity showing a reduced thickness of the left abdominal wall muscle and quadratus lumborum. (A) Coronal image. (B) Axial image. (C) Reduced cross-sectional area of the left quadratus lumborum.

The patient was assigned to the Department of General Surgery because her major symptoms included abdominal discomfort and bulging after meals. Computed tomography (CT) of the abdomen and pelvic cavity showed atrophic changes in the abdominal wall muscles (including the external oblique, internal oblique, and transversus abdominis muscles) on the left side without intestinal hernia (Fig. 2A, B). The thickness of the left abdominal wall muscle was reduced by 30% to 40 % compared to the right side in the coronal and axial images. Moreover, the cross-sectional area of the adjacent quadratus lumborum muscle on the left side was less than that on the right side at the L4 level axial plane (Fig. 2C). A general surgery specialist confirmed that the surgical procedure for the correction of abdominal muscle weakness was not indicated. Consequently, the patient was referred to our pain clinic for specialized pain management.

**Figure 3.**
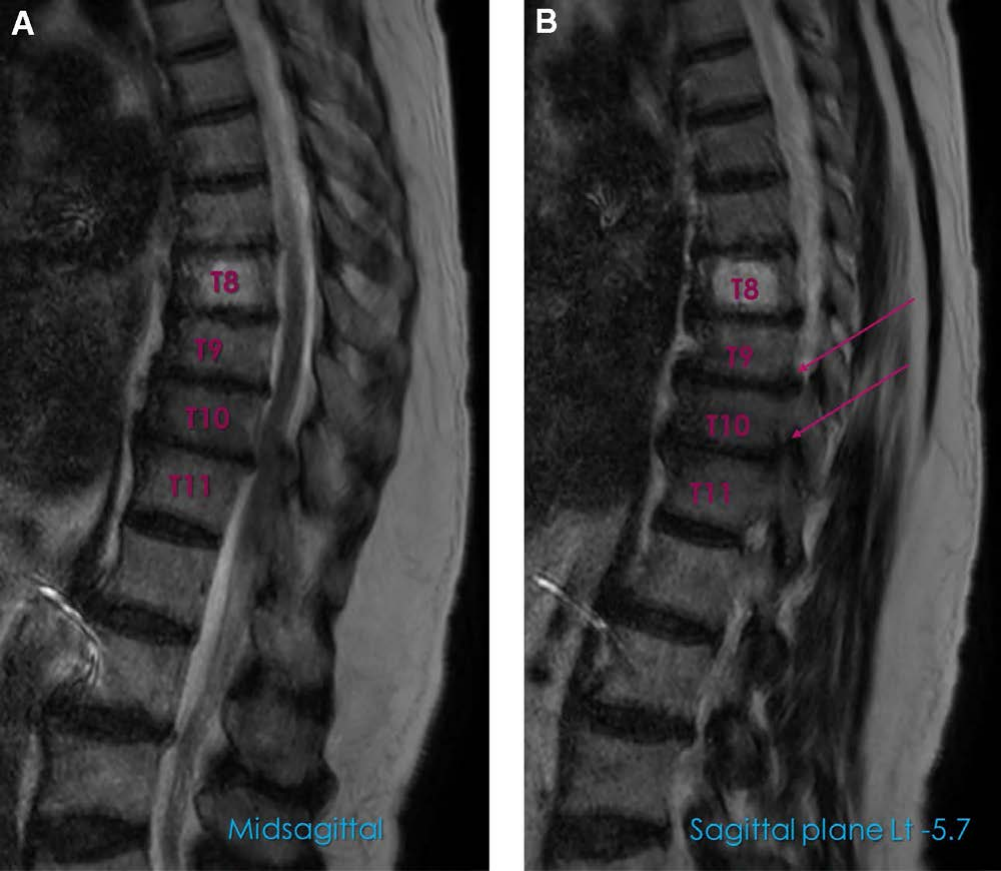
Sagittal whole-spine magnetic resonance T2-weighted imaging showing multilevel HTD at T8/9/10/11. (A) Midsagittal plane. (B) Sagittal plane suggesting foraminal herniated disc at left T9/10/11. HTD = herniation of the thoracic intervertebral disc.

**Figure 4.**
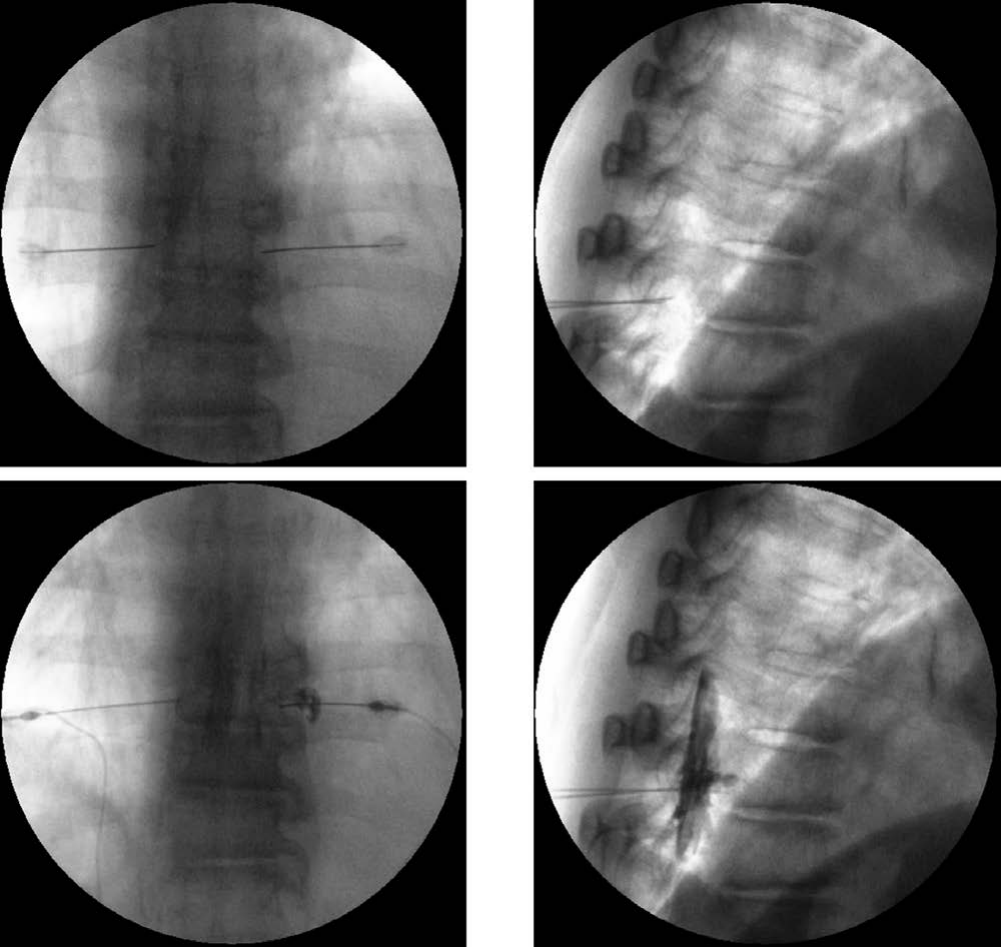
Thoracic transforaminal epidural block performed at the T10/11 level in case 1.

Based on the physical and radiological findings, a pain specialist performed the transversus abdominis plane block or quadratus lumborum block several times to exclude the possibility of truncal neuropathy following muscular atrophy. Trigger point injections in the quadratus lumborum and abdominal wall muscles, where a positive local twitch response was identified, were performed concomitantly. However, symptomatic relief was temporary, and the patient’s abdominal muscle weakness persisted for months. The patient underwent sagittal whole-spine magnetic resonance imaging (MRI) for further evaluation. Based on this evaluation, she was diagnosed with multiple HTD at T8/9/10/11 without myelopathy. A foraminal herniated disc at the left T9/10/11 was also observed (Fig. 3A, B). Subsequently, transforaminal epidural block was performed once per week (Fig. 4). Following 3 epidural blocks, the patient reported pain reduction of 50%, and the pain intensity was decreased from 6 to 3 on the NRS. She was advised to use analgesic medications as required and encouraged to modify lifestyle risks, such as weight reduction. The patient was subsequently lost to follow-up.

### 2.2. Case 2

A 75-year-old man visited our pain clinic because of aggravated postherpetic neuralgia (PHN) 1 month prior to presentation. Herpes zoster was diagnosed in the left T12 dermatome approximately 7 months ago, and the patient was treated with antiviral medication during the acute stage at a local clinic. However, he was referred to our pain clinic because of persistent PHN 3 months later. Peripheral nerve blocks, such as intercostal nerve block, rather than epidural block, were planned to minimize the possibility of intraspinal hemorrhage because he was taking direct oral anticoagulants because of his recent history of pulmonary thromboembolism. The zoster-related pain markedly improved after the second peripheral nerve block. The patient was prescribed analgesics for symptomatic relief. However, 3 months later, the patient revisited our clinic with recurrent aggravated PHN for 1 month. The patient stated that he had received pulsed radiofrequency (RF) treatment for PHN at another tertiary care hospital; however, this failed to relieve his symptoms.

We planned to use thermal RF for the treatment of the refractory pain. On preprocedural examination, there were no sensory abnormalities, including hypesthesia, hyperalgesia, or allodynia. However, according to the patient, the previously symptomatic area extended to the upper dermatome (most likely to be T10 and T11). During the first diagnostic block before the RF procedure, the dorsal root ganglion (DRG) block for T11 and an additional T10 produced temporary symptomatic relief. The results of the second diagnostic block for T12 (original herpes zoster-related symptomatic area) were similar. During the RF procedure, a combination of thermal and pulsed RF procedures was performed. Thermal RF was performed only at the T12 DRG because this area produced strong concordant pain during sensory and motor stimulation. Pulsed RF treatment of the T11 and T10 DRG, which showed mild concordant pain during nerve stimulation, was added.

**Figure 5.**
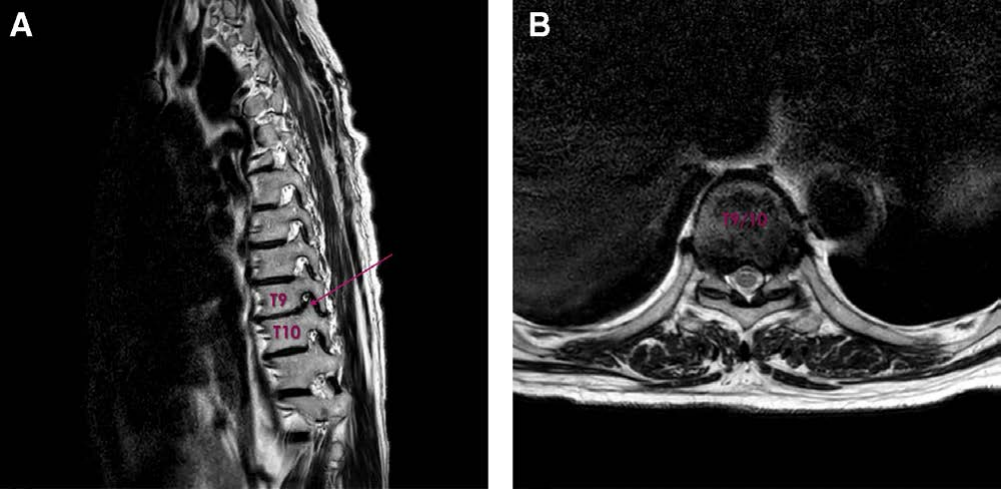
Magnetic resonance T2-weighted imaging of thoracic spine showing the foraminal herniated disc at the left T9/10 level. (A) Sagittal plane. (B) Axial plane.

One month later, hypesthesia of the T12 dermatome was confirmed using a cold swab. Sustained throbbing and aching pain at the T9/10/11 dermatomal distribution was noted. A T-spine MRI was performed to identify possible reasons. A foraminal herniated disc at the left T9/10 level with a mild degree of disc protrusion at T8/9/10 was identified, which was compatible with his current symptoms (Fig. 5A, B). A transforaminal epidural block at T9/10/11 was performed once per week. After the second block, the patient reported a symptomatic relief of from 9 to 5 on the NRS. At the 3-month follow-up, the patient’s pain had markedly improved with analgesic medications alone.

## 3. Discussion

HTD is a rare disease that accounts for <1% of all disc herniations.^[1]^ The clinical features of HTD are highly variable,^[6]^ and the localization of the involved thoracic level can be more confusing than that of the cervical or lumbar spine. The differential diagnosis of HTD is extensive and may include several spinal and nonspinal causes.^[8]^ Therefore, physicians often experience diagnostic errors because of their clinical complexity and scarcity. HTD may be misunderstood as a lumbar spinal pathology because it may cause leg symptoms suggestive of lumbar radiculopathy.^[9]^ More severe forms of HTD with accompanying cord compression may also display vague leg pain, paraplegia, hyperactive deep tendon reflexes, and positive ankle clonus or Babinski response.^[10]^ Early diagnosis and prompt surgical treatment are crucial for preserving and restoring function.

In this case report, the authors present 2 patients with unusual HTD in different clinical situations. In case 1, the patient had unusual mimicking symptoms, with the chief complaint of abdominal discomfort and bulging aggravated after meals rather than pain. Therefore, the patient was primarily evaluated for the possibility of operable causes, such as abdominal hernia, by using abdominal CT. Regretfully, the authors did not focus on abdominal wall muscle atrophy, which was a consequence of denervation changes derived from more proximal pathology. The authors initially thought that the abdomen enlargement and abdominal wall muscle atrophy may have stretched the abdominal cutaneous nerve, leading to nerve irritation, due to the “push” mechanism in developing nerve entrapment syndrome.^[11,12]^ Moreover, positive direct tenderness and local twitch responses in the quadratus lumborum and adjacent abdominal wall muscles further led to an incorrect diagnosis.

In case 2, symptomatic thoracic radiculopathy due to HTD was misinterpreted as PHN. It is well known that herpes zoster generally involves a single DRG unilaterally, although multiple ganglia involvement has been reported in herpes zoster developing in the cranial nerve, such as in Ramsay Hunt syndrome.^[13,14]^ Therefore, physicians should always check the level of herpes zoster involvement in a single dermatome, especially for guiding interventional pain management. However, currently used dermatomal maps are variable and inaccurate.^[15]^ The reason for this variability is due to the skin area being innervated by 2 or more spinal roots, and the presence of an intersegmental anastomosis between the spinal nerve roots.^[16]^ This phenomenon confounded the diagnosis in case 2. This case highlights the importance of careful history-taking and physical examination. This may include determining the nature of the pain and characteristic relieving or aggravating factors, such as the Valsalva maneuver, to discriminate radiculopathy from other pathologies.

In addition to diagnostic difficulty, HTD has therapeutically challenging issues owing to the relatively higher complication rates of surgical treatment.^[17]^ In this case report, both patients were successfully treated with epidural block. Several guidelines for lumbar and cervical disc herniations suggest a favorable level of evidence for transforaminal epidural steroid injections in radiculopathies.^[18,19]^ For HTD, there are no consensus guidelines, and the literature on the topic is sparse.^[20,21]^ Further investigations and evidence-based consensus guidelines are necessary. Our 2 patients experienced considerable symptomatic relief of a 50% reduction in NRS following thoracic transforaminal epidural blocks with steroid or hypertonic saline. We identified the appropriate contrast flow reaching the nerve root and the anterior epidural space using fluoroscopy. For the epidural block to be effective, the drugs must reach the anterior epidural space because the disc–nerve interface is located there. Target-specific epidural blocks were therapeutically effective, and HTD and accompanying radiculopathy were considered the main causes of patient’ complaints.

## 4. Conclusion

Multilevel HTD of the mid- to lower-thoracic spine may present as abdominal bulging with atrophy of the abdominal wall muscles. Concomitant PHN and HTD at adjacent thoracic levels may also occur. Thoracic transforaminal epidural block should be considered as a conservative therapeutic approach for HTD.

## Author contributions

Data curation : Yu Jin Oh and Seon Hwa Nam

Methodology : A Ram Doo and Cheol Jong Woo

Writing- original draft : Min Jong Ki

Writing- review & editing : A Ram Doo
